# Effect of Environmental Exposure to Zearalenone on the Metabolic Profile of Patients with Sigmoid Colorectal Cancer or Colorectal Cancer on the Day of Hospital Admission

**DOI:** 10.3390/ijms26146967

**Published:** 2025-07-20

**Authors:** Sylwia Lisieska-Żołnierczyk, Magdalena Gajęcka, Łukasz Zielonka, Katarzyna E. Przybyłowicz, Maciej T. Gajęcki

**Affiliations:** 1 Independent Public Health Care Center of the Ministry of the Interior and Administration and the Warmia and Mazury Oncology Center in Olsztyn, Wojska Polskiego 37, 10-228 Olsztyn, Poland; lisieska@wp.pl; 2 Department of Veterinary Prevention and Feed Hygiene, Faculty of Veterinary Medicine, University of Warmia and Mazury in Olsztyn, Oczapowskiego 13, 10-718 Olsztyn, Poland; lukasz.zielonka@uwm.edu.pl (Ł.Z.); gajecki@uwm.edu.pl (M.T.G.); 3 Department of Human Nutrition, Faculty of Food Sciences, University of Warmia and Mazury in Olsztyn, Słoneczna 45F, 10-719 Olsztyn, Poland; katarzyna.przybylowicz@uwm.edu.pl

**Keywords:** zearalenone mycotoxicosis, sigmoid colorectal cancer, colorectal cancer, metabolic profile

## Abstract

Colorectal cancer is one of the most commonly diagnosed types of cancer and constitutes the second most frequent cancer in women (W) and the third most frequent cancer in men (M). The aim of the study was to determine if environmental exposure to zearalenone (ZEN) (a mycoestrogen) affects the metabolic profile of patients diagnosed with sigmoid colorectal cancer (SCC) and colorectal cancer (CRC) (division based on their location) at hospital admission. Male and female patients who were diagnosed with SCC or CRC and whose blood samples tested positive or negative for ZEN participated in a year-long study. Seventeen patients with symptoms of SCC and CRC, in whom ZEN and its metabolites were not detected in peripheral blood, constituted the patients without ZEN (PWZ) group. The experimental groups comprised a total of 16 patients who were diagnosed with SCC or CRC and tested positive for ZEN but negative for ZEN metabolites. Patients exposed to ZEN were characterized by increased levels of liver enzymes (alanine aminotransferase (ALT) from 5.8 to 18.1 IU/L; aspartate aminotransferase (AST) from 2.8 to 10.7 IU/L) and decrease in the value of the De Ritis ratio (below 1.0), different gamma glutamyl transpeptidase and AST activity, lower albumin (from 0.24 g/dL in M to 0.67 g/dL in W) and total protein levels (from 0.75 to 1.76 g/dL), a decrease in total cholesterol (from 21.6 to 40.3 mg/dL) and triglyceride levels (from 7.8 to 37.2 mg/dL), and lower activity of lipase C (from 28.72 to 64.75 IU/L). The metabolic profile of M and W patients diagnosed with SCC and CRC and exposed to ZEN revealed intensified biotransformation processes in the liver, liver damage, and a predominance of catabolic processes.

## 1. Introduction

In cancer biology, metabolic reprogramming is recognized as a hallmark of tumor development, reflecting how cancer cells adapt to environmental stressors, modify energy use, and alter nutrient pathways. These metabolic shifts support cell proliferation, influence immune responses, and remodel the tumor microenvironment [1,2,3].

Colorectal cancer, including its sigmoid variant, can arise spontaneously or as a complication of chronic intestinal inflammation. This inflammation is highly prevalent worldwide and considered a major public health concern [4]. It is often triggered by dysbiosis of the intestinal microbiota [5,6] and metabolic disruptions that create a pro-carcinogenic environment within the intestinal mucosa. Chronic inflammation is a physiological condition caused by long-term tissue trauma that leads to cellular changes and an immune response, resulting in the repair of damaged tissue and cell proliferation in place of damaged tissue [7,8]. In turn, the pathogenesis of inflammation of the intestinal mucosa consists of many stages after the initiation of the inflammatory process, during which normal mucosal epithelium is transformed to reveal abnormal foci in crypts, adenoma, atypical hypertrophy, and carcinogenesis [9,10].

In this context, it is increasingly important to understand how environmental toxins, such as ZEN, contribute to the cascade of biochemical and metabolic changes that accompany or accelerate tumorigenesis. The liver, as the primary site of xenobiotic metabolism, plays a crucial role in detoxifying such compounds. However, the biotransformation of mycotoxins can also lead to the generation of reactive metabolites that are equally or more toxic than the parent compound [11,12,13,14].

Zearalenone is a fat-soluble, non-steroidal mycotoxin with an estrogenic character (mycoestrogen), produced by *Fusarium* molds that colonize crops such as maize, wheat, barley, oats, and rice. Due to its structural similarity to estrogens, ZEN can bind to estrogen receptors in various organs, including the liver and gastrointestinal tract [15,16]. ZEN is highly stable under food storage conditions and resistant to processing, which increases the risk of dietary exposure [17]. Once ingested, ZEN may impair immune function, modulate inflammatory responses, and interfere with DNA integrity [18,19]. It has been classified as a Group 3 carcinogen by the IARC, based on limited evidence in animals and inadequate evidence in humans [20].

The gastrointestinal tract is the first point of direct contact between the macroorganism and mycotoxins [20]. ZEN disrupts intestinal architecture and barrier function, leading to local inflammation and increasing systemic vulnerability to toxicants [9,21,22]. This mechanism is particularly relevant to SCC and CRC, where long-term inflammatory conditions may be amplified by additional environmental exposures such as mycotoxins [18,23].

The cancer microenvironment is shaped not only by intrinsic mutations but also by extrinsic agents, including contaminants like ZEN, which act as environmental co-factors in carcinogenesis [24]. Cells—both healthy [25] and cancerous—respond to such stressors by undergoing metabolic shifts, often reflected in alterations in liver function, protein metabolism, lipid profiles, and enzymatic activity [2,6,26].

Routine laboratory blood tests provide critical insights into organ function and systemic homeostasis. Metabolomic analyses offer a comprehensive overview of an individual’s physiological state, allowing the detection of subtle disruptions that may be linked to external factors like dietary toxins [27,28,29]. Despite their diagnostic potential, such analyses are still underutilized, in part due to the complexity of interpretation and the need for interdisciplinary knowledge. Changes in liver enzyme activity, protein synthesis, lipid metabolism, and other biochemical markers may serve as indicators of early hepatic dysfunction and systemic inflammation conditions that are often exacerbated by mycotoxins. The serum metabolic profile is a valuable clinical tool for detecting such dysfunctions [30,31].

The aim of the study was to investigate whether there is a relationship between the presence of ZEN and/or its metabolites in food materials (naturally contaminated) and the metabolic profile results of patients (W and M) with SCC or CRC on the day of hospital admission.

## 2. Results

The presented results were obtained as part of a comprehensive study examining the potential effects of food-borne ZEN on the progression of carcinogenesis in the distal sections of the large intestine in W and M [13,14].

### 2.1. Concentrations of ZEN and Its Metabolites

The concentration of ZEN (parent substance) differed considerably across patients. Zearalenone metabolites, α-ZEL and β-ZEL, were not identified in any group (Table 1).

Zearalenone and its metabolites were not detected in the PWZ group (Table 1) (the results may have been below the sensitivity of the method). No significant differences were found in the SCC group, and the difference between the mean values noted in W and M reached 4.93 ng/mL. In the CRC group, the difference between the mean values noted in W and M was significant (74.53 ng/mL; *p* ≤ 0.05). No clinical signs of zearalenone mycotoxicosis were observed during hospitalization.

### 2.2. Metabolic Reprogramming During SCC and CRC [2]

#### 2.2.1. Liver Function Test

**Figure 1:**
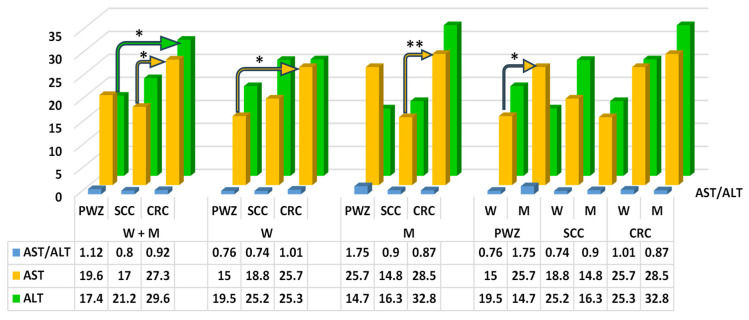
Mean concentrations (±) of the De Ritis ratio (AST/ALT), aspartate aminotransferase (AST, IU/L) and alanine aminotransferase (ALT, IU/L) in the peripheral blood of women (W) and men (M) from PWZ (patients without zearalenone), SCC (sigmoid colorectal cancer), and CRC (colorectal cancer) groups. Differences are significant at * *p* ≤ 0.05 and ** *p* ≤ 0.01.

The mean values of ALT differed significantly (*p* ≤ 0.05) between patients (W + M) from PWZ and CRC groups (difference of 12.2 IU/L) (Figure 1). Reference values according to Roche (RvaR), the range is 2–33 IU/L for W and 2–41 IU/L for M.

The mean values of AST differed significantly (*p* ≤ 0.05) between patients (W + M) from SCC and CRC groups, between W from PWZ and CRC groups, and between W and M in the PWZ group (difference of 10.3 IU/L, 10.7 IU/L, and 10.7 IU/L, respectively). A highly significant difference (*p* ≤ 0.01) was noted only between M from SCC and CRC groups (difference of 13.7 IU/L) (Figure 1).

No significant differences were noted in the values of the De Ritis ratio (Figure 1), which could be attributed to considerable variations in SD values in each of the study groups.

**Figure 2:**
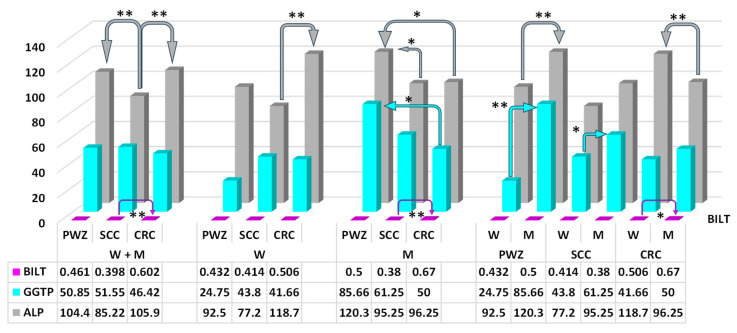
Mean concentrations (±) of total bilirubin (BILT—mg/dL), gamma glutamyl transpeptidase (GGTP, IU/L) and alkaline phosphatase (ALP, IU/L) in the peripheral blood of women (W) and men (M) from PWZ (patients without zearalenone), SCC (sigmoid colorectal cancer), and CRC (colorectal cancer) groups. Differences are significant at * *p* ≤ 0.05 and ** *p* ≤ 0.01. between W from SCC and CRC groups (difference of 41.5 IU/L), between W and M in the PWZ group (difference of 27.8 IU/L), and between W and M in the CRC group (difference of 22.45 IU/L).

Significant differences (*p* ≤ 0.05) in ALP values were found between W from the PWZ group vs. SCC and CRC groups (difference of 25.05 IU/L and 24.05 IU/L, respectively) (Figure 2). Highly significant differences (*p* ≤ 0.01) were noted between patients (W + M) from the SCC group vs. PWZ and CRC groups (difference of 19.18 IU/L and 20.68 IU/L, respectively).

Significant differences (*p* ≤ 0.05) in GGTP values (Figure 2) were observed between M from PWZ and CRC groups (difference of 35.66 IU/L), and between W and M in the SCC group (difference of 17.45 IU/L). Highly significant differences (*p* ≤ 0.01) were found between W and M in the PWZ group (difference of 60.91 IU/L).

The mean values of BILT (Figure 2) differed significantly between W and M in the CRC group (difference of 0.17 mg/dL). Highly significant differences in this parameter were noted between W + M and M from SCC and CRC groups (difference of 0.204 mg/dL and 0.29 mg/dL, respectively).

#### 2.2.2. Comprehensive Metabolic Panel

The comprehensive metabolic panel supports early detection of metabolic disorders that primarily affect the conversion of energy and other compounds.

**Figure 3:**
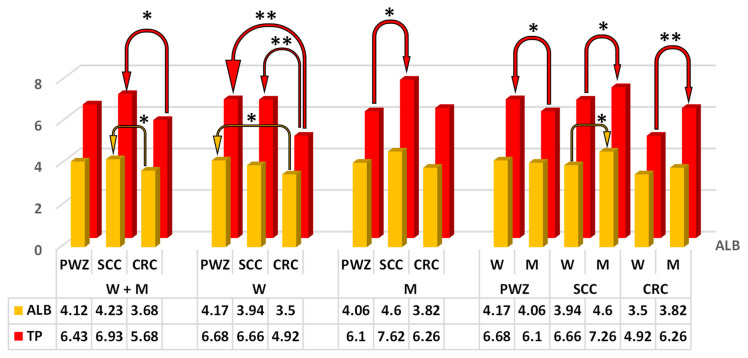
Mean concentrations (±) of albumin (ALB, g/dL) and total protein (TP, g/dL) in the peripheral blood of women (W) and men (M) from PWZ (patients without zearalenone), SCC (sigmoid colorectal cancer), and CRC (colorectal cancer) groups. Differences are significant at * *p* ≤ 0.05 and ** *p* ≤ 0.01.

Significant differences (*p* ≤ 0.05) in mean TP values (Figure 3) were observed between W + M from SCC and CRC groups (difference of 1.25 g/dL), between M from PWZ and SCC groups (difference of 1.52 g/dL), and between W and M in PWZ and SCC groups (difference of 0.58 g/dL and 0.6 g/dL, respectively).

Highly significant differences (*p* ≤ 0.01) in TP levels were found between W from the CRC group vs. PWZ and SCC groups (difference of 1.76 g/dL and 1.74 g/dL, respectively), and between W and M in the CRC group (difference of 1.34 g/dL).

The mean values of ALB differed significantly (*p* ≤ 0.05) (Figure 3) between patients (W + M) from SCC and CRC groups (difference of 0.55 g/dL), between W from PWZ and CRC groups (difference of 0.67 g/dL), and between W and M in the SCC group (difference of 0.66 g/dL).

**Figure 4:**
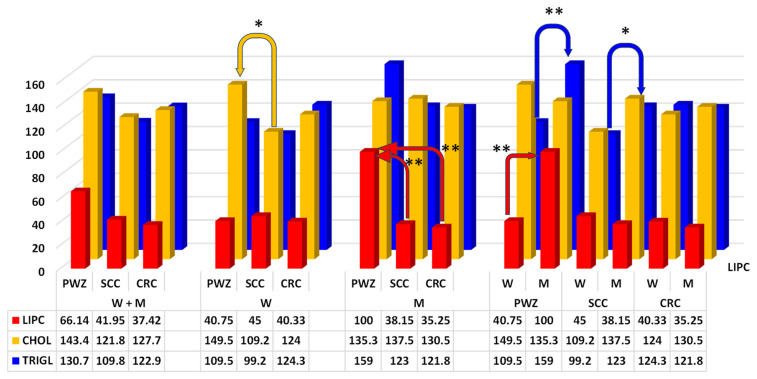
Mean concentrations (±) of lipase C (LIPC, IU/L), total cholesterol (CHOL, mg/dL), and triglycerides (TRIGL, mg/dL) in the peripheral blood of women (W) and men (M) from PWZ (patients without zearalenone), SCC (sigmoid colorectal cancer), and CRC (colorectal cancer) groups. Differences are significant at * *p* ≤ 0.05 and ** *p* ≤ 0.01.

Only highly significant differences (*p* ≤ 0.01) in LIPC activity (Figure 4) (RvaR—13–60 IU/L) were noted between M from the PWZ group vs. SCC and CRC groups (difference of 61.85 IU/L and 64.75 IU/L, respectively).

The mean values of CHOL (Figure 4) (RvaR—115–190 mg/dL) differed significantly (*p* ≤ 0.05) between W from PWZ and SCC groups (difference of 40.3 mg/dL).

An analysis of TRIGL levels (Figure 4) (RvaR—74–106 mg/dL) revealed significant differences (*p* ≤ 0.05) between W and M in the SCC group (difference of 23.8 mg/dL), and highly significant differences (*p* ≤ 0.01) between W and M in the PWZ group (difference of 49.5 mg/dL).

**Figure 5:**
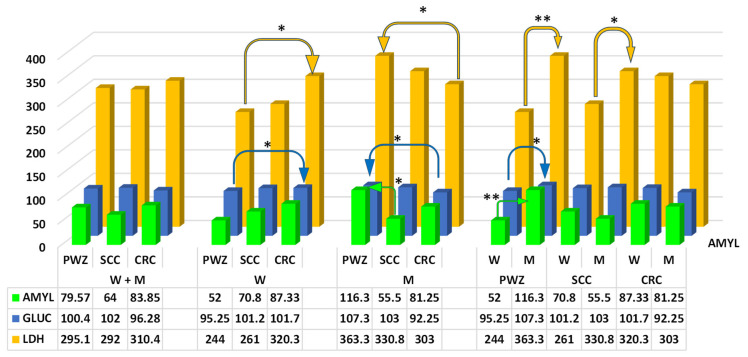
Mean concentrations (±) of amylase (AMYL, IU/L), glucose (GLUC, mg/dL), and lactate dehydrogenase (LDH, IU/L) in the peripheral blood of women (W) and men (M) from PWZ (patients without zearalenone), SCC (sigmoid colorectal cancer), and CRC (colorectal cancer) groups. Differences are significant at * *p* ≤ 0.05 and ** *p* ≤ 0.01.

The values of AMYL (Figure 5) (RvaR—28–100 IU/L) differed significantly (*p* ≤ 0.05) between M from PWZ and SCC groups (difference of 60.8 IU/L). A highly significant difference (*p* ≤ 0.01) in the analyzed parameter was noted between W and M in the PWZ group (difference of 119.3 IU/L).

The values of GLUC (Figure 5) (RvaR—74–106 mg/dL) differed significantly (*p* ≤ 0.05) between W and M from PWZ and CRC groups (difference of 6.45 mg/dL and 15.05 mg/dL, respectively) and between W and M in the PWZ group (difference of 12.05 mg/dL).

The values of LDH (Figure 5) (RvaR—240–480 IU/L) differed significantly (*p* ≤ 0.05) between W and M from PWZ and CRC groups (difference of 76.3 IU/L and 60.3 IU/L, respectively) and between W and M in the SCC group (difference of 69.8 IU/L). A highly significant difference in LDH values was observed between W and M in the PWZ group (difference of 119.3 IU/L).

## 3. Discussion

Under normal circumstances, the body maintains the functions and stability of the internal environment through three major metabolic pathways (amino acids, glucose, and lipids) that are reprogrammed after the onset of cancer and are further involved in maintaining the changes provoked by tumor microenvironments [2,26]. The results of this study suggest that in patients with SCC and CRC, cells can undergo discreet reprogramming [32] in response to environmental stressors such as ZEN [29,33], thus acquiring specific metabolic properties.

In male and female patients with SCC and CRC who were naturally exposed to ZEN and its metabolites, the results of the liver function test and the comprehensive metabolic panel are difficult to interpret because no such studies have been undertaken to date.

### 3.1. Zearalenone

In the present study, ZEN levels varied between patients with symptoms of SCC and CRC, but considerable differences were noted in SD values (Table 1), which could explain the small number of significant differences. Interestingly, ZEN metabolites, α-ZEL and β-ZEL, were not detected (the obtained values were below the sensitivity of the method), which could be attributed to “increased demand” for ZEN, an exogenous compound with high estrogenic activity [18], in postmenopausal [34] and andropausal patients [35]. Absolute and relative estrogen levels decline and fluctuate considerably in postmenopausal women. Therefore, exposure to a substance with high estrogenic activity, such as ZEN, contributes to uncontrolled changes in metabolic processes [36]. These changes promote metabolic reprogramming, which, in conditions such as SCC or CRC, can affect the duration of cell proliferation [2,37]. In CRC, metabolic changes are driven by intense anaerobic glycolysis (Warburg effect), changes in lipid and amino acid metabolism, thus contributing to tumor growth, invasion, and metastasis [26]. This observation suggests that prolonged exposure to ZEN (mycoestrogen, xenoestrogen, or an undesirable substance) could be associated with cancerous lesions in the gastrointestinal tract [18], as evidenced by the results of the comprehensive metabolic panel in this study, as well as the results of other studies analyzing the levels of steroid hormones [13,38] and thyroid hormones [14].

Zearalenone was not identified in CRC or SCC patients in the PWZ group (indicating that carcinogenesis does not have to be accompanied by zearalenone mycotoxicosis), possibly because this mycotoxin was absent in plant-based foods consumed by the studied individuals or because the concentration of this undesirable substance was below the sensitivity of the method.

Zearalenone is metabolized in the liver, but it also exerts toxic effects on this multifunctional gland [18]. Gajęcka et al. [16] and Wu et al. [39] confirmed the hypothesis that ZEN is immunoreactive towards ERs and affects the expression of genes encoding liver enzymes that participate in the biotransformation and neutralization of undesirable substances. In turn, Llorens et al. [40] argued that ZEN is also able to generate free radicals in a dose-dependent manner and modify the expression of antioxidant enzymes and oxidative stress-related genes both in vivo and in vitro [41]. Zearalenone levels were very high (Table 1) in the blood of patients diagnosed with SCC and CRC (229.12 ng/mL and 214.61 ng/mL, respectively). This observation was made by comparing mycotoxin levels in omnivorous animals, such as prepubertal gilts exposed to 5, 10, and 15 μg of ZEN/kg body weight for 42 days [42]. The values noted in the current study were 100 times higher. The above suggests that the examined patients were exposed to higher doses of ZEN, which led to hepatotoxic effects and a subsequent decrease in the effectiveness of detoxification processes.

The presented arguments indicate that ZEN is a compound with multifaceted biological activity [13,14,34], which affects its concentrations in peripheral blood. Zearalenone’s activity is also apparent based on the results of the liver function test, which evaluates the excretory and metabolic activity of the liver and potential damage to liver cells.

### 3.2. Metabolic Reprogramming Effect in Patients with SCC and CRC

Cancerous changes in the large intestine (SCC or CRC) are most often caused by genetic, environmental, or lifestyle risk factors. Zearalenone and its metabolites are potential environmental risk factors [18]. In pre-pubertal gilts exposed to low doses of ZEN for 42 days, this mycotoxin increased the expression of ER-β in the descending colon [15], altered the abundance and species composition of the microbial community [21,43], and caused its shift to the distal segment of the large intestine. Zearalenone mycotoxicosis contributes to changes in gut microbial activity during the progression and recurrence of colorectal cancer, and the development of therapy resistance [5]. Therefore, it has been hypothesized that in SCC and CRC patients, a potential interaction [44] exists between the presence of ZEN in peripheral blood and selected blood biochemical parameters. The interplay between selected metabolic indicators and carcinogens, such as ZEN [35], should be further explored to increase our understanding of abnormalities in the metabolic profile. In inflammatory states, metabolic and systemic disorders are associated with the progression and medical prognosis of colorectal cancer (SCC and/or CRC). The survival rate of patients with CRC varies considerably, and the existing predictive models should be urgently modified to account for environmental factors, such as climate change, which increases the number of carcinogens or undesirable substances in food.

Aspartate aminotransferase (AST) and alanine aminotransferase (ALT)—Despite the fact that ZEN and its metabolites target mainly tissues in the reproductive system, these mycotoxins are biotransformed primarily by the liver and the intestines [18]. Liver function tests elucidate the potential causes of clinical symptoms, and they enable physicians to monitor disease progression and assess liver damage [45]. In the present study, the activity of both AST and ALT was higher (Figure 1) in patients exposed to ZEN than in the PWZ group. The above was accompanied by an increase in GGTP activity (Figure 2) in W and a decrease in GGTP activity in M in all study groups, and this parameter was always higher in men (significant difference) (the normal reference range is <35 IU/L for women and <60 IU/L for men). The levels of ALT (Figure 1) were within the RvaR range (5–40 IU/L). This parameter ranged from 29.6 to 32.8 IU/L only in M in the CRC group, and it was determined at 17–19 IU/L in the PWZ group (Figure 1). In turn, AST activity (RvaR range: 5–40 IU/L) was higher in M in the CRC group than in the PWZ group in the majority of cases (Figure 1), and the observed differences were statistically significant, which points to inflammation and cancer progression [46,47].

The observed increase in ALT and AST activity could be attributed to liver cell damage during the biotransformation of ZEN, which leads to the release of these enzymes into the bloodstream. The values of both enzymes are usually higher in men than in women because of differences in body weight. However, it should also be noted that ALT and AST activity were highest in the CRC group. Balló et al. [35] demonstrated that in men, exposure to ZEN leads to hepatotoxicity, immunotoxicity, genotoxicity, carcinogenicity, intestinal toxicity, reproductive toxicity, and endocrine disruptions, thus contributing to obesity during andropause. In other mammalian species, ZEN induced a significant increase in the blood levels of AST, ALT, and GGTP (differences between the sexes were not analyzed), which are reliable markers of liver damage [39].

De Ritis ratio—Serum biomarkers determined in routine clinical tests are widely used to diagnose cancer, determine the prognosis, and the risk of recurrence because these tests are non-invasive, cheap, and easy to perform. These biomarkers include AST and ALT—enzymes that are produced by both cancerous and non-cancerous cells and are released into the bloodstream. Alanine aminotransferase is present mainly in the liver, while AST is present in various tissues, including the liver, heart, skeletal muscles, and kidneys.

The ratio of AST to ALT is referred to as the De Ritis ratio [48,49], and it is an independent prognostic factor. The De Ritis ratio is a widely recognized biochemical marker with significant applications in the diagnosis and treatment of various diseases, especially liver disorders and cancer.

Abnormal serum levels of liver enzymes could point to differences in metabolic processes, but in the tumor microenvironment. In a study by Fu et al. [50], the values of the De Ritis ratio were not affected by the patients’ age or gender, tumor location, or tumor diameter. This observation suggests that the De Ritis ratio alone is a reliable parameter for predicting the outcome of stage II/III non-metastatic colorectal cancer. The cited authors concluded that the De Ritis ratio could play a key role in predicting the course of CRC, adjusting treatment strategies, and ultimately improving patient outcomes. The De Ritis ratio should exceed 1 [51] because AST activity is always higher than ALT activity in healthy subjects. Fu et al. [50], Scheipner et al. [48], Wang et al. [52], and Bezen et al. [53] found that high values of the AST/ALT ratio were associated with a worse prognosis in non-metastatic cancers. A preoperative increase in the AST/ALT ratio to more than 1.26 is an independent prognostic factor for both metastasis-free survival and overall survival. In the current study, this parameter exceeded 1.26 only in the mean in the PWZ group (Figure 1). In the groups where ZEN was present in the blood serum of patients with SCC and CRC, this situation could be attributed to the fact that liver cells were additionally involved in ZEN biotransformation [15]. As a result, the release of ALT from the liver was intensified, as shown by the comparison of ALT levels in W and M from the PWZ group and SCC and CRC groups (Figure 1). The latter corroborates the observations made by Fu et al. [50].

Total bilirubin (BILT)—Chronic inflammatory bowel disease is one of the hallmarks and antecedents of intestinal cancer [54], but it also accompanies mycotoxicoses [7,9], and the released inflammatory cells can also produce reactive oxygen species, which leads to mutations in cancer cells [55]. Experimental and clinical studies have shown that serum bilirubin (a novel metabolic hormone—[56]) is a by-product of hemoglobin breakdown with significant anti-inflammatory and antioxidant properties [57,58]. Men tend to have higher total bilirubin levels than women (Figure 2) because of lower estrogen levels [13] and higher red blood cell counts. In addition, the effect of sex hormones on the activity of certain transferases and differences in the degree of their expression between W and M lead to differences in the conjugation of bilirubin genes and circulating levels of bilirubin between the sexes, which may partly explain the gender-related differences in the risk of CRC, observed in this study. The even greater difference between W and M in the current study was associated with the ongoing zearalenone mycotoxicosis, i.e., the presence of a mycoestrogen in the patients’ organisms (Table 1; Figure 2). Higher levels of circulating bilirubin were positively associated with the risk of bowel cancer in M in all groups, especially in the CRC group (Figure 2).

Gamma glutamyl transpeptidase (GGTP)—Both GGTP and AST are markers of liver damage, but their presence in the blood serum is associated with environmental or genetic factors. Gamma glutamyl transpeptidase binds to the cell membrane, participates in amino acid transfer and glutathione metabolism, and plays a key role in the antioxidant processes provoked by ZEN [59]. This observation is validated by the results noted in W, who probably had a much higher “demand” for ZEN than M, which is why ZEN was more easily biotransformed in W than M (Figure 2). In turn, AST is a cytoplasmic or mitochondrial enzyme that catalyzes the transfer of amino acid groups in the gluconeogenesis and metabolism of amino acids. The observed differences in GGTP and AST levels indicate that metabolic processes such as ZEN biotransformation were enhanced [15] or that inflammatory processes in the gastrointestinal tracts were provoked by the presence of ZEN that was ingested with contaminated foods or recovered in the second stage of detoxification [18], which increased retention time. These findings suggest that GGTP could be a marker of cancer, especially CRC [47], regardless of its stage.

Alkaline phosphatase (ALP)—Advances in molecular technology have highlighted the importance of ALP as a key diagnostic marker (Figure 2) for selecting the most effective therapeutic strategy in CRC patients [60]. Alkaline phosphatase levels are usually elevated in patients with CRC, which was observed only in W in the present study. However, a comparison of ALP activity in W and M revealed an increase in PWZ and SCC groups, and a decrease in M in the CRC group. The noted values are within the upper reference range (RvaR: 30–120 IU/L). However, notably, the present study involved postmenopausal or andropausal patients. On the other hand, ALP may inhibit the inflammatory response to the presence of ZEN in the intestinal lumen [7], but this enzyme is also involved in tumor growth. However, ALP levels have never been compared in patients with SCC or CRC, especially individuals exposed to ZEN. This study revealed significant differences (*p* ≤ 0.01) between W and M from PWZ and CRC groups, but the reasons for these discrepancies remain unknown. Perhaps, ALP activity increases (upper reference range) in response to tumor growth rather than mycotoxicosis.

Albumin (ALB)—Albumin is a key nutritional indicator (Figure 3). Cancer cells often have a higher metabolic demand for ALB, which decreases ALB retention and contributes to hypoalbuminemia [60]. In the present study, ALB values were in the upper reference range (RvaR: 3.5–5.5 g/dL), and no significant differences in this parameter were observed. The above indicates that the patients had adequate nutritional status without any contraindications to further therapy or surgery, and that ZEN mycotoxicosis had no effect on ALB as a prognostic marker. According to some authors, elevated ALB values are associated with lower ALP levels in cancer patients [61], but this observation was not confirmed in the present study.

Total protein (TP)—The nutritional status of cancer patients is an important predictor of survival [62]. Nutritional status is assessed based on selected blood biochemical parameters (total protein, albumin, lymphocyte counts, and total cholesterol), which are not influenced by subjective factors and can serve as quick and objective indicators of the patient’s nutritional status and immunity. Total protein levels (Figure 3) in the blood are influenced by the synthesis (anabolism) and degradation (catabolism) of two main protein fractions: albumin (Figure 3) and globulins. In turn, serum cholesterol levels support reliable assessments of the patient’s nutritional and immune status (Figure 4). Liver damage (reduced synthesis) or cancer are the most common causes of abnormally low plasma protein levels (hypoproteinemia) [23]. There is evidence to indicate that the metabolic characteristics of cancer cells change as the disease progresses, and typical metabolic changes include dysregulated amino acid absorption, increased nitrogen requirements, and increased utilization of anabolic metabolic pathways. Metabolic reprogramming may be useful in diagnosing early stages of cancer. An analysis of blood samples collected upon hospital admission revealed significant differences between W and M from SCC and CRC groups vs. the PWZ group, probably because of differences in the levels of endogenous estradiol [13] and mycoestrogen (Table 1), especially in W from the CRC group. These findings are confirmed by the values of prognostic markers (free triiodothyronine/free thyroxine—fT3/fT4), which were indicative of advanced neoplastic changes in all patients from SCC and CRC groups [13]. Both arguments prove that the metabolic processes essential for cancer progression and inflammation (tumor microenvironment) proceed much more rapidly in women [63], probably in response to ZEN exposure.

Triglycerides (TRIGL), total cholesterol (CHOL) and lipase C (LIPC)—Lipids are important diagnostic markers, and research has shown that cancer cells induce changes in lipid metabolism. Proliferating cancer cells exhibit unique metabolic patterns that enable them to obtain sufficient energy to synthesize proteins and nucleic acids. The above leads to structural changes in cell membranes, disruptions in energy homeostasis, cell signaling, gene expression and protein distribution, which influences cell functions such as apoptosis, autophagy, necrosis, proliferation, or differentiation. In SCC and CRC, metabolic reprogramming triggers changes in lipid levels, including TRIGL [64]. Triglycerides (Figure 4) store highly concentrated metabolic energy, which is released from adipose tissue in the form of fatty acids and transported via the blood to target tissues. Yang et al. [65] that increased serum or plasma levels of TRIGL were associated with an increased risk of colorectal adenoma. This relationship appears to be stronger in the colon than in the rectum, and in M than in W. A similar increase in TRIGL values was noted in the current study, especially in M, regardless of the study group. Triglyceride levels ranged from 121 to 159 mg/dL (Figure 4) and exceeded the RvaR reference range. However, TRIGL values were much higher in the PWZ group (not exposed to ZEN) at 159 mg/dL. In patients who tested positive for ZEN on the day of hospital admission, TRIGL values were determined at 123 mg/dL (SCC group) and 121.8 mg/dL (CRC group). These findings suggest that ZEN slows down the release of concentrated energy from adipose tissue into the blood [23] or that ZEN biotransformation processes also require energy from lipids [15]. However, inflammatory bowel disease can increase the risk of CRC via a different mechanism, such as zearalenone mycotoxicosis. This observation stems from the fact that cancer cells [66] and inflammatory processes in the lumen of the gastrointestinal tract need lipids to proliferate [7]. In addition, some fatty acid metabolites act as signaling molecules to maintain a balance between pro- and anti-inflammatory signals [64]. Li et al. [67], Fang et al. [68] and Sever et al. [69] demonstrated that lower levels of CHOL and higher levels of TRIGL (dyslipidemia markers) are associated with a higher risk of CRC. Similar observations were made in the present study (Figure 4). Interestingly, CHOL and TRIGL values were lower in SCC and CRC groups (W + M) than in the PWZ group, which could suggest that tumor growth requires more energy during zearalenone mycotoxicosis.

When CHOL levels are low, sterol regulatory element binding proteins (SREBPs) are transported to the Golgi complex, where they are processed to release an active fragment that can enter the nucleus, bind to SREBPs, and regulate the expression of the genes and enzymes participating in CHOL synthesis. Cholesterol uptake increases, and the production of endogenous CHOL is also enhanced in many cancer cells [70]. De novo synthesis of CHOL is intensified because of the overexpression of the key enzymes and transcription factors involved in the CHOL biosynthetic pathway. Notably, inflammation has long been recognized as a key factor in the initiation and progression of cancer [71]. Serum CHOL levels can decrease in advanced stages of disease because lipids can provoke tumor recurrence by providing the energy for the growth and proliferation of tumor cells.

The role of LIPC in CRC cells remains unclear [72]. In the present study, LIPC values were within the RvaR reference range in SCC and CRC groups (Figure 4). These values were much higher in the PWZ group, and in M they exceeded the upper reference range (RvaR) by approximately 66.6%. This result could indicate that ZEN exerts anticarcinogenic effects or that the studied patients were deficient in lipids that act as a source of energy.

Glucose (GLUC), amylase (AMYL), and lactate dehydrogenase (LDH) (Figure 5)—Luo et al. [73] and Zhao and Wu [74] observed a relationship between blood glucose levels and the risk of CRC. An increased risk of CRC could be associated with the direct effects of high glucose levels (within a specific range of values—[75]. The above generates metabolic benefits for cancer cells and the associated factors, including: (i) oxidative stress in cancer cells [33,76], and (ii) inflammation, especially in the gastrointestinal tract [9,54]. In addition, hyperglycemia drives tumor cell proliferation, migration, and invasion, while inhibiting apoptosis. Supportive factors include inflammatory processes that are exacerbated by hyperglycemia. Inflammation is the immune system’s response to cellular damage, infection, or other stimuli. However, chronic inflammation can lead to tissue damage and cancer [74]. Mycotoxins, especially ZEN and its metabolites, have been recognized as pro-inflammatory factors [7,9,75]. The relationship between diabetes mellitus and CRC is clinically significant in this context. It can be hypothesized that while diabetes is an independent risk factor for CRC [77], its negative effects on the gastrointestinal tract may be exacerbated by ZEN. This hypothesis is validated by the results of the present study (Figure 5), where glucose levels were within the upper reference range (RvaR) and were only slightly higher in one M in the PWZ group (107.3 mg/dL). In addition, the mean glucose levels in groups (regardless of gender) were highest in the SCC group and slightly lower in the CRC group. These observations suggest that high levels of glucose (within the upper reference range) and ZEN (which causes intestinal inflammation) increase the risk of CRC.

Amylase is a digestive enzyme and an endocrine biomarker that is secreted in response to stress. Elevated AMYL levels are associated with increased mental and physical stress [78]. Recent research has shown that amylase activity can also increase in the initial stages of exposure to mycotoxins [33,79]. In the present study, AMYL levels (Figure 5) were within the upper reference range (RvaR). According to Sasaki et al. [46], AMYL levels are associated with pancreatic exocrine and endocrine dysfunction, which could increase the risk of CRC by modulating the gut microbiota. A decrease in *Enterococcus faecalis* counts could be indicative of CRC. In turn, Cieplińska et al. [21] reported that the size of microbial populations (especially *Escherichia coli* and *Enterococcus faecalis*) in the distal segment of the large intestine increased significantly in pre-pubertal gilts in response to much lower ZEN levels than those noted in the present study. Stress and ZEN induced clear inflammatory changes in the intestinal mucosa [9]. In the current study, AMYL levels were within the upper reference range (RvaR) (Figure 5) and were slightly higher than 100 IU/L only in M from the PWZ group. It appears that the relationship between ZEN activity and AML activity remains unclear in SCC and CRC. The fact that AMYL levels were higher in M could be attributed to the fact that postmenopausal women have a higher demand for ZEN than andropausal men.

Lactate dehydrogenase is regarded as a prognostic factor in patients with CRC. This enzyme plays an important role in cellular metabolism and energy production. Lactate dehydrogenase is found in all bodily tissues, red blood cells, liver, skeletal muscles, kidneys, and the heart. Lactate dehydrogenase reprograms glucose metabolism and intensifies the Warburg effect, thus contributing to the onset and progression of CRC. This molecular mechanism significantly influences CRC initiation and progression [80]. This observation was confirmed by Mowat and Al-Abada [81], who found that cancer cells proliferate in an uncontrolled manner, consume large amounts of oxygen in their microenvironment, and rely on anaerobic glycolysis to derive the energy for proliferation and survival. The high expression of LDH is closely related to tumor progression, tumor size, and the degree of cell differentiation. In this experiment, LDH values were within the lower reference range (RvaR) (Figure 5) in all groups. This parameter exceeded 300 IU/L only in M and in all patients with CRC. However, LDH is not a specific biomarker for cancer. This parameter reflects tissue damage, including in injuries and diseases that are not related to cancer [82].

## 4. Materials and Methods

### 4.1. Patients

#### Inclusion Criteria

A single-center, case-control study was conducted based on the medical history of W and M patients with SCC or CRC. The main inclusion criterion was the presence of ZEN and/or its metabolites in the patients’ blood. The inclusion criteria on the day of hospital admission were histologically confirmed SCC or CRC, age of 54 years and older, absence of organ dysfunctions, and a score of 0 or 1 on the Eastern Cooperative Oncology Group (ECOG PS) performance scale. The exclusion criteria were an insufficient number of patients with histologically confirmed SCC and CRC, and cancers other than stage II and III colorectal cancer diagnosed using the tumor-node-metastasis (TNM) cancer staging system [32]. Geriatric changes affecting tissue or organ function in older (postmenopausal or andropausal) patients were taken into consideration because these processes can affect the interaction between liver activity and the ongoing “low-dose” zearalenone mycotoxicosis (adaptive effect—[83]).

The study was conducted in accordance with the provisions of the World Medical Association’s Declaration of Helsinki (Ethical Principles for Medical Research in Human Subjects) and Ethics for Researchers: Facilitating Research Excellence in FP7. No samples or patients were lost during the study, but the reasons for the absence of ZEN metabolites in blood samples have not been fully elucidated. This result could be attributed to the rapid biotransformation of ZEN [84] caused by physiological estrogen deficiency in postmenopausal and andropausal patients.

### 4.2. Study Design

Blood samples for laboratory analyses were collected from patients who were hospitalized with a histologically confirmed diagnosis of SCC and CRC. The participants signed informed consent forms and were informed that they could deny access to their personal and medical information at any time during the study. All medical records were anonymized. The participants were patients of the Independent Public Healthcare Center of the Ministry of the Interior and Administration and the Warmia and Mazury Oncology Center in Olsztyn, Poland. The participants were residents of the voivodeship of Warmia and Mazury. The study lasted one year.

The study involved all W and M who were admitted to the hospital with a diagnosis of cancer (SCC or CRC) and who tested positive or negative for ZEN. Data were obtained from laboratory analyses and tests performed before hospitalization (because of natural biological detoxification lasting up to 72 h—[85]). Zearalenone and its metabolites were quantified by the Department of Veterinary Prevention and Feed Hygiene of the Faculty of Veterinary Medicine, University of Warmia and Mazury in Olsztyn, Poland. Selected parameters in the blood of patients with histologically confirmed SCC and CRS were determined by the Independent Public Healthcare Center of the Ministry of the Interior and Administration and the Warmia and Mazury Oncology, Poland.

The participants were divided into two study groups. The first group consisted of patients whose blood samples tested negative for ZEN and/or its metabolites (PWZ group). The second group comprised patients whose blood samples tested positive for ZEN (experimental group). Each group was further divided into subgroups based on gender and location of cancer.

The PWZ group consisted of 17 patients (8 W and 9 M) who accounted for 51.5% of the study population and tested negative for ZEN and its metabolites α-zearalenol and β-zearalenol (α-ZEL and β-ZEL, respectively). In the PWZ group, 8 patients (4 W and 4 M) had been diagnosed with SCC, and 9 patients (4 W and 5 M) had been diagnosed with CRC. The mean age was 61.5 years in W and 68.3 years in M.

The experimental group consisted of 16 patients (8 W and 8 M) who accounted for 48.5% of the study population and had been diagnosed with malignant tumors in the distal part of the large intestine. These patients tested positive for ZEN only (parent mycotoxin). In this group, 9 patients (5 W and 4 M) had been diagnosed with SCC (SCC group, 27.3% of the study population), and 7 patients (3 W and 4 M) had been diagnosed with CRC (CRC group, 21.2% of the study population). In the SCC group, the mean age was 65.8 years in W and 70.2 years in M. In the CRC group, the mean age was 54.3 years in W and 70.5 years in M.

### 4.3. Blood Sampling

Blood samples for the quantification of ZEN, α-ZEL, β-ZEL, ALT, AST, alkaline phosphatase (ALP), gamma glutamyl transpeptidase (GGTP), total bilirubin (BILT), total protein (TP), albumin (ALB), lipase C (LIPC), total cholesterol (CHOL), triglycerides (TRIGL), amylase (AMYL), glucose (GLUC), and lactate dehydrogenase (LDH) were collected from SCC and CRC group patients on the day of hospital admission using the method described by Guder et al. [86]. Blood was collected between 7:00 and 9:00 a.m., after a 12 h fasting period and 1 h after smoking the last cigarette, in a relaxed atmosphere, before diagnostic and therapeutic procedures. Blood was collected using Vacuette Quickshield Safety Tube Holders (Greiner Bio-One GmbH, Kremsmunster, Austria). Blood samples of 20 mL each were collected from the basilar vein or the median antecubital vein into EDTA-K2 tubes (Vacuette Tube, Greiner Bio-One GmbH, Kremsmunster, Austria).

The separated plasma for the determination of ZEN, α-ZEL, and β-ZEL was stored at −18 °C until analysis.

Blood for biochemical analyses was not stored. The tubes were tightly sealed, stored upright, protected from light, and had limited contact with air. The samples were transported to the laboratory within one hour after collection. The samples were centrifuged at 3000 rpm for 20 min at 4 °C. The following blood biochemical parameters were determined: ALT, AST, ALP, GGTP, and BILT in the liver function test, and TP, ALB, LIPC, CHOL, TRIGL, GLUC, LDH, and AMYL in the comprehensive metabolic panel.

### 4.4. Extraction Procedure and Quantification of ZEN, α-ZEL, and β-ZEL

The presence of ZEN, α-ZEL, and β-ZEL in the blood plasma was determined using immune affinity columns (Zearala-TestTM Zearalenone Testing System, G1012, VICAM, Watertown, MA, USA). All extraction procedures were carried out in accordance with the manufacturers’ instructions. After extraction, the eluents were placed in a water bath with a temperature of 40 °C and evaporated in a stream of nitrogen. Subsequently, 0.5 mL of acetonitrile (LCMS grade) was added to the dry residue to dissolve the mycotoxin. The concentrations of ZEN, α-ZEL, and β-ZEL in the blood plasma were determined with the use of an Agilent chromatographic system (Santa Clara, CA, USA) comprising a G7167A multisampler, G7104C quaternary pump, G7116A multicolumn thermostat, and 6470 double quadrupole LC/TQ mass spectrometer. The prepared samples will be analyzed with the use of the Zorbax rapid resolution chromatographic column (2.1 × 50 mm; 1.8 micron Agilent Eclipse Plus C18) in gradient mode. The mobile phase will contain 0.1% (*v*/*v*) formic acid in water (solvent A) and 0.1% (*v*/*v*) formic acid in acetonitrile (solvent B). Gradient conditions will be as follows: initially, 20% B that increases to 100% B in 4.0 min and back to 20% B in 0.1 min.

Mycotoxin concentrations were determined with an external standard and were expressed in ppb (ng/mL). Matrix-matched calibration standards were used in the quantification process to eliminate matrix effects that can potentially reduce sensitivity. The calibration standards were dissolved in matrix samples using the same procedure that was applied to prepare the remaining samples. The calibration material was free of mycotoxins. The detection limits for ZEN, α-ZEL, and β-ZEL were defined as the concentration at which the signal-to-noise ratio decreases to 3. The concentrations of ZEN, α-ZEL, and β-ZEL were determined in all groups. The LOQ will be estimated as the triple LOD value (Table 2). The specificity of the method will be determined by comparing the chromatograms of a blank sample with those corresponding to a spiked tissue sample.

The mass spectrometer was operated with ESI in the negative ion mode. The MS/MS parameters were optimized for each compound. The linearity was tested by a calibration curve including six levels. Table 2 shows the optimized analysis conditions for the mycotoxins tested.

### 4.5. Quantification of Blood Biochemical Parameters

Blood biochemical parameters, including (i) ALT, AST, De Ritis ratio, ALP, GGTP, and BILT in the liver function test, and (ii) TP, ALB, LIPC, CHOL, TRIGL, GLUC, LDH, and AMYL in the comprehensive metabolic panel, were determined using colorimetric, enzymatic, and immunoturbidimetric assays in the Cobas Integra 400 Plus analyzer (Roche, Basel, Switzerland).

### 4.6. Statistical Analysis

The results were processed statistically at the Department of Discrete Mathematics and Theoretical Computer Science of the Faculty of Mathematics and Computer Science, University of Warmia and Mazury in Olsztyn, Poland. The bioavailability of ZEN and its metabolites and the examined blood biochemical parameters were determined in both experimental groups and in the PWZ group. In each sample, the results were expressed as means (x^−^) with standard deviation (SD). The following parameters were evaluated: (i) differences in mean values (W + M) between PWZ, SCC, and CRC groups; (ii) differences in the mean values of W and M diagnosed with SCC or CRC; and (iii) differences in the mean values of W and M within groups (PWZ, SCC, and CRC). All differences were determined by one-way ANOVA. If the differences between group means were statistically significant, the differences between pairs of means were determined using Tukey’s multiple comparison test. The homogeneity of variance across groups was assessed using Levene’s test and the Brown–Forsythe test. If the hypothesis of equal variance was rejected in both tests, the significance of differences was assessed using the Kruskal–Wallis test. In all tests, the results were considered highly significant at *p* < 0.01 (**) and significant at 0.01 < *p* < 0.05 (*). All data were processed in Statistica v. 13.3.0 (TIBCO Software Inc., Silicon Valley, CA, USA).

## 5. Conclusions

This study is the first to demonstrate that environmental exposure to zearalenone (ZEN) may significantly contribute to metabolic alterations in patients with sigmoid colorectal cancer (SCC) and colorectal cancer (CRC). The presence of ZEN in peripheral blood was associated with measurable disruptions in liver function and lipid metabolism, suggesting hepatocellular stress and intensified catabolic activity. In particular, patients exposed to ZEN exhibited elevated liver enzyme activity, reduced albumin and total protein levels, and lower concentrations of cholesterol and triglycerides compared to non-exposed individuals.

The data support the hypothesis that environmental mycotoxins such as ZEN may influence cancer-related metabolic reprogramming and create a biochemical milieu conducive to disease progression. These findings highlight the need to consider environmental xenobiotics as potential modulators of cancer biology and encourage further exploration of ZEN’s impact on metabolic homeostasis in oncological patients.

## Figures and Tables

**Table 1:** Mean concentrations (±) and standard deviation (SD) of ZEN and its metabolites (α-ZEL and β-ZEL) (ng/mL) in the peripheral blood of patients from all groups (* *p* ≤ 0.05).

Groups	ZEN		α-ZEL		β-ZEL	
Patients	W	M	W	M	W	M
**PWZ**	0	0	0	0	0	0
**SCC**	224.19 ± 98.96	229.12 ± 84.02	0	0	0	0
**CRC**	289.14 * ± 38.72	214.61 ± 144.75	0	0	0	0

**Key:** 0—below the sensitivity of the method.

**Table 2:** Optimized conditions for mycotoxins tested.

Analyte	Precursor (*m*/*z*)	Production (*m*/*z*)	Fragmentor Voltage (V)	Collision Energy (eV)	LOD (ng mL^−1^)	LOQ (ng mL^−1^)	Linearity (%R^2^)
ZEN	317.1	273.3 187.1	160	25 33	0.03	0.1	0.999
*α*-ZEL	319.2	275.2 160.1	144	21 33	0.3	0.9	0.997
*β*-ZEL	319.2	275.2 160.1	144	21 33	0.3	1	0.993

## Data Availability

Due to the sensitive nature of the questions asked in this study, survey respondents were assured that raw data would remain confidential and would not be shared.
